# COVID-19 ethnic inequalities in mental health and multimorbidities: protocol for the COVEIMM study

**DOI:** 10.1007/s00127-022-02305-y

**Published:** 2022-06-23

**Authors:** E. Impara, I. Bakolis, L. Bécares, H. Dasch, A. Dregan, J. Dyer, M. Hotopf, R. J. Stewart, R. Stuart, J. Ocloo, J. Das-Munshi

**Affiliations:** 1 Department of Psychological Medicine, Institute of Psychiatry, Psychology and Neuroscience, King’s College London, De Crespigny Park, London, SE5 8AF UK; 2 University of Sussex, Brighton, UK; 3 Black Thrive Global, NHS-E/I, London, UK; 4 Centre for Implementation Science, Health Services, Population and Research Department, Institute of Psychiatry, Psychology and Neuroscience (IoPPN), King’s College London, London, UK; 5 National Institute for Health Research (NIHR) Applied Research Collaboration South London (NIHR ARC South London) At King’s College Hospital NHS Foundation Trust, London, UK; 6 South London and Maudsley NHS Foundation Trust, London, UK; 7 Department of Biostatistics and Health Informatics, Institute of Psychiatry, Psychology and Neuroscience, King’s College London, London, UK

**Keywords:** COVID-19, Ethnicity, Severe mental illness, Mortality, Service use

## Abstract

**Purpose:**

The COVID-19 pandemic may have exacerbated ethnic health inequalities, particularly in people with multiple long-term health conditions, the interplay with mental health is unclear. This study investigates the impact of the pandemic on the association of ethnicity and multimorbidity with mortality/service use among adults, in people living with severe mental illnesses (SMI).

**Methods:**

This study will utilise secondary mental healthcare records via the Clinical Record Interactive Search (CRIS) and nationally representative primary care records through the Clinical Practice Interactive Research Database (CPRD). Quasi-experimental designs will be employed to quantify the impact of COVID-19 on mental health service use and excess mortality by ethnicity, in people living with severe mental health conditions. Up to 50 qualitative interviews will also be conducted, co-produced with peer researchers; findings will be synthesised with quantitative insights to provide in-depth understanding of observed associations.

**Results:**

81,483 people in CRIS with schizophrenia spectrum, bipolar or affective disorder diagnoses, were alive from 1st January 2019. Psychiatric multimorbidities in the CRIS sample were comorbid somatoform disorders (30%), substance use disorders (14%) and personality disorders (12%). In CPRD, of 678,842 individuals with a prior probable diagnosis of COVID-19, 1.1% (*N *= 7493) had an SMI diagnosis. People in the SMI group were more likely to die (9% versus 2% in the non-SMI sample) and were more likely to have mental and physical multimorbidities.

**Conclusion:**

The effect of COVID-19 on people from minority ethnic backgrounds with SMI and multimorbidities remains under-studied. The present mixed methods study aims to address this gap.

## Background

The SARS-CoV-2/COVID-19 pandemic, declared by the World Health Organization (WHO) in 2020 as a public health emergency of international concern (PHEIC) [1], has exacerbated pre-existing health inequalities [2]. In the UK, people living in more deprived areas have had higher COVID-19 diagnosis and death rates compared to individuals who live in less deprived locations [3]. COVID-19 admissions to intensive care were over-represented by minority ethnic groups who are more likely to die [4], with Black and Asian communities registering the highest death rates from COVID-19 [3]. This can be attributed to a variety of inter-related factors: multimorbidity [2], healthcare access inequalities, discrimination and socioeconomic disadvantage [5] and more recently, reports that healthcare management approaches are sub-optimal and potentially biased for these groups [6].

Research has established that pre-existing long-term chronic conditions are strong predictors of COVID-19-related mortality [7, 8]. Their presence has been cited as one reason why ethnic health inequalities may have been exacerbated as a result of COVID-19 [9]. This is a complex relationship to disentangle, with a number of factors cited, including an over-representation of minority ethnic people in lower paid precarious employment, or being less able to work from home, and being more likely to have been occupationally exposed to COVID-19 infection (e.g. transport, healthcare and hospital cleaning), as well as residing in over-crowded households in deprived neighbourhoods [10].

The impact of COVID-19 on people with mental disorders and multimorbidities remains unclear. This is a major public health concern since people with mental disorders were already known to die 15–20 years earlier than the general population [11], mostly from preventable conditions [12], with minority ethnic groups also vulnerable [12]. COVID-19 has led to significant changes in primary care and secondary mental healthcare provision, including an acute drop in face-to-face contacts and discharges [13]. At the same time, public health messages initially discouraged people from ‘unnecessary’ contacts with healthcare [2]. These messages may have disproportionately impacted on minority people [2], a group whose experiences of inequality in healthcare have been overlooked. Finally, with vaccine roll-out now established in many countries, concerns around uptake and barriers to access among certain groups have also been raised, but the impact of this remains unclear.

Previous research attempting to assess ethnic inequalities in healthcare have been hampered by a lack of conceptual clarity. Standard data collection approaches also lead to smaller sample sizes for ethnic minority participants which can also lead to analytic challenges. In addition, there are concerns that marginalised people are not adequately involved in the research process itself. In this project, we aim to tackle these challenges through using an innovative mixed methods design, working with large-scale electronic health records, representative of local ethnically diverse populations, as well as with nationally representative samples from the UK. Quantitative insights will be further informed by qualitative research with data collection co-produced with people with lived experience of using mental health services, who also identify as being of a minority ethnic background. We anticipate that the participatory action research approach of this work will allow an interrogation of the following research questions [14]:

1. How has COVID-19 exacerbated ethnic health inequalities (in service use/ care pathways and mortality) in adults with both severe and common mental disorders and physical health multimorbidities?
2. What are the mechanisms (e.g. differential withdrawal of services impacting minority ethnic patients, changes to health-seeking, or anticipated or experienced discrimination) through which any inequalities identified in research question 1 have been perpetuated?
3. How may findings (from research questions 1 and 2) inform co-production of actionable recommendations, so that further inequalities can be prevented, and patient safety improved as the pandemic (including societal and economic consequences) progresses?

## Methods

### Patient/stakeholder involvement

Central to our approach is our collaboration with individuals who are involved in the Patient and Carer Race Equality Framework (PCREF) initiative. The PCREF is a framework which is being implemented in England, through NHS-England, and which seeks to directly tackle race inequalities in mental health. PCREF national approaches are to be informed by findings at three ‘pilot’ sites; these are Manchester, Birmingham & Solihull, and Lambeth (in southeast London). Sites for recruitment to the qualitative study (as described below) have been identified on this basis.

Alongside lived experience researchers being a part of the research team and contributing to data collection and analysis, we will also convene a steering group comprising people with lived experience of mental illness, as well as clinical and academic representatives from each of the three study sites. The steering group will assist with advising on study design (e.g. ethics submission, topic guides and sampling/recruitment strategies) as well as contextualising findings with local insights and also assist in co-producing summaries and actionable recommendations for the study.

### Quantitative approaches

To assess the objectives of the study we will use two large-scale datasets, selected to complement each other. Datasets to be used will include the Clinical Record Interactive Search system (CRIS), consisting of mental healthcare records from a large secondary mental healthcare Trust in London, and the Clinical Practice Research Database (CPRD), electronic health records from primary care across England. Whereas CRIS provides naturalistic ethnically ‘boosted’ samples from southeast London [15] with the ability to provide in-depth analyses relating to the impact of COVID-19 on people living with mental disorders, CPRD is nationally representative [16], permitting an assessment of national/regional trends and a closer assessment of physical health comorbidities. We will assess if local findings from CRIS converge/diverge with the national context in England (CPRD).

### Datasets

#### The Clinical Record Interactive Search system (CRIS)

CRIS is a platform permitting free text/structured field searches of de-identified electronic health records (EHRs) [15]. CRIS covers all electronic health records (EHRs) from South London & Maudsley (SLaM) Trust, one of Europe’s largest secondary mental healthcare providers, providing secondary mental healthcare to 1.3 million residents [15]. Half of SLaM service users are of a minority ethnic background, with Black Caribbean, Black African, Indian, Pakistani, Bangladeshi, and Irish people represented [12].

#### Participants and measures from CRIS

A cohort of 81,483 people with confirmed schizophrenia-spectrum and affective disorders will be derived from CRIS, for the study. Within the CRIS system, mental health diagnoses are clinician-ascribed, according to the *International Classification of Mental Disorders-10 (ICD-10)* [17] and captured through free-text and structured fields [15]. For the purposes of this study, ‘severe mental illnesses’ (SMI) will include people receiving a schizophrenia-spectrum disorder diagnosis (ICD-10 code: F2*), or bipolar affective disorder diagnosis (ICD-10: F30*/F31*). In addition, affective disorder diagnoses (ICD-10 code F3*) will also be included, as at the level of secondary mental healthcare, these may be considered more ‘severe’. The cohort will comprise all persons with an SMI or affective disorders diagnosis, alive on 1st January 2019 followed until death or end of the study (5th May 2021). Additional data on age, gender, ethnicity (mapped on to ONS census ethnicity groups: White British, Irish, Black Caribbean, Black African, Indian, Pakistani, Bangladeshi), marital status, area-level deprivation through the Index of Multiple Deprivation (IMD) at lower super output level, linked to patient postcode [18], other psychiatric comorbidities (according to ICD-10 criteria for each chapter) will be used to inform analyses and our definition of multimorbidity: this involves the presence of two or more mental health conditions. These conditions are: schizophrenia and affective disorder, anxiety and somatoform disorders, eating disorders, pervasive developmental disorders, learning disabilities, personality disorders, dementia and substance use disorders, which will permit a closer assessment of psychiatric multimorbidities [19]. Psychiatric diagnoses according to ICD-10 chapter will be captured through the structured fields as well as through Natural Language Processing (NLP) approaches which have previously been validated and additionally help to identify under-recorded co-occurring diagnoses [15].

#### Outcomes in CRIS

Data on consultations (remote or face to face) and mental health service use (e.g. admissions under the Mental Health Act or in-patient admissions without any prior contact with community teams) will be used for the analysis.

Data on all-cause mortality are available through weekly reports whereby the Mental Health Trust is notified of deaths (with date of death occurrence) of service users who are either currently or ever active under the care of the Trust. Data on cause-specific mortality will be available through a linkage to Office for National Statistics (ONS) mortality data, providing cause of death derived from death certificates and dates for death occurrence and registration.

#### Overview of data from primary care in England: Clinical Practice Research Database (CPRD)

The Clinical Practice Research Database (CPRD) is the world’s largest database of primary care EHRs and is nationally representative [16]. Use of the CPRD dataset will enable an assessment of how ethnic inequalities in people living with multimorbidities and mental disorders have been exacerbated as a result of COVID-19, with a specific focus on primary care data. As 98% of the population is registered with primary care in the UK, with the general practitioner usually being the initial port of call for any health concern, use of this dataset will provide important insights, at population level, with good detail on the presence of physical and mental health multimorbidities.

Prospective data from about 1400 general practices across the United Kingdom from 1990 to present, are available. CPRD includes information on psychiatric and physical health diagnoses, specialist referrals, treatments, COVID-19 status, (testing/clinical symptoms) and mortality. Around 80% of patients in CPRD have ethnicity recorded. Given the impact of COVID-19 nationally which has shown strong regional variation, trends in infection, service use and mortality by region will be explored.

### Participants and measures from CPRD

A cohort of 678,842 people with positive COVID-19 tests, including a subset of people with ‘probable’ COVID-19 diagnoses (based on SNOMED medical codes provided by the CPRD) will be developed. Within this cohort, there are 7493 people with severe mental illnesses, defined as the presence of schizophrenia-spectrum disorders, bipolar disorders, and non-organic psychosis. In addition, the presence of physical multimorbidities will be determined by the co-existence of one or more chronic physical disorders^1^ (heart disease, hypertension, kidney disease, cancer, diabetes, asthma, psoriasis, etc.). These conditions will be identified using SNOMED medical codes developed by our team (AD). Other variables to be used in the analysis will include age, gender as well as ethnicity, mapped on to Census criteria (White British, Irish, Black Caribbean, Black African, Indian, Pakistani, Bangladeshi), age, area-level deprivation (assessed through the Index of Multiple Deprivation (IMD) linked to lower layer Super Output Level Area (LSOA) based on the patient’s post code of residence. ‘Region’ will be assessed by NHS/government regions, including London, South-East, East of England, West Midlands, South-West, Wales, East Midlands. There is also the opportunity to link CPRD data to urban and rural classification data, both at patient (England only) and practice level.

### Outcomes in CPRD

Date of death is available in CPRD and will be used to denote ‘death from any cause’. To assess “consultations”, we will assess average number of consultations per month per patient (remote or face to face).

### Statistical analyses

#### CRIS

We will model the patients’ survival rate via Cox Proportional Hazards Regression. Survival time will be modelled as a function of age, sex, ethnicity and psychiatric multimorbidities. As we are interested in the effect of the declaration of a global pandemic on this relationship, an interaction term for time (since declaration of the pandemic as a PHEIC by WHO) will be included in our model. We will also use Regression Discontinuity Designs. This quasi-experimental design will allow the possibility of establishing the effect on an intervention (e.g. lockdown) by allocating a threshold above/below which the intervention is assigned: in this way, the design recreates conditions similar to those of a control group [20].

#### CPRD

Multi-level Cox Proportional Hazards Regression models (MLMs) will be developed, specifying associations at the individual-level nested in General Practices, further nested in English regions. MLMs will be used to assess the association of exposures/covariates with all-cause mortality outcomes. Entry into the cohort for analysis will be latest of the time of (suspected or confirmed) diagnosis of COVID-19 infection or current registration date. End of the study will either be date of death, transferred out date, or study end date. A priori confounders will include age, gender and area level deprivation.

First, to assess whether people living with SMI are at an increased risk of death due to (confirmed or suspected) COVID-19 infection, we will compute crude and adjusted Hazard Ratios for death (from time of confirmed/ suspected COVID-19 infection) in people with severe mental illness compared to people without severe mental illnesses in the cohort. We will assess whether the risk of death in people with severe mental illnesses was modified by ethnicity. Crude and adjusted estimates will be presented stratified by ethnicity. The ‘multimorbidities’ variable will be added to models to enable an assessment of how far observed mortality differences were accounted for by the presence of multimorbidities in people also diagnosed with SMI. Finally, ‘total number of consultations (remote and/ or face-to-face)’ to assess how far this may have also confounded estimates for the association of SMI*ethnicity with risk of death. If sample sizes permit further breakdown we will assess estimates also stratified by region and use the ICC (intracluster correlation coefficient) across MLMs to assess variability of estimates within and between regions, in the analysis. Strength of associations will be assessed using Likelihood ratio tests or Wald tests as appropriate.

Analyses across datasets will be conducted in R (4.1.1) [21] and STATA 13 [22].

#### Statistical power

For analyses involving the CRIS cohort, with a known SMI sample of people in the White British ethnicity group (*N *= 32,324) and 5.7% deaths, and known sample sizes of each of the minority ethnic groups within the study (including *N *= 2326 for an aggregated South Asian group (comprising Indian, Pakistani, Bangladeshi people), the study will have sufficient power (> 80%) to detect differences in risk ratios for all-cause mortality, ranging from 0.6 to 1.4 for the smallest group (other ethnicity, *N *= 826) and risk ratios ranging from 0.9 to 1.1 for the largest group (Black Caribbean *N *= 10,466).

For analyses involving the CPRD cohort, assuming a sample size of people without SMI of *N *= 671,349 with 2% deaths, and an SMI group of *N *= 7493 with 9% deaths, given the large number of patients within each of the minority ethnic groups (ranging from *N *= 8129 in the smallest (mixed ethnicity) group to *N *= 243,181 in the largest (other ethnicity) group, our study will have sufficient power (90%) to detect even small differences in risk ratio of 0.9 to 1.1 for all-cause mortality between different ethnic groups.

### Qualitative approaches

To address the qualitative aims of the project, we will use a participatory action research (PAR) framework [23], that is also drawing upon “insider–outsider” perspectives of people with lived experience of the issues under investigation [24]. A PAR approach is appropriate when seeking to address problems in an organisation and make improvement [23, 25]. PAR is particularly relevant to the development of co-produced research with patients, the public and communities as it is a methodology that seeks to challenge the dominance and control of how knowledge is defined and create more equal partnerships in the research process [23, 26, 27]. Researchers with lived experience in mental health service use, who identify as being of a minority ethnic background, will be recruited through the community partnership for Black mental health, Global Black Thrive, PCREF study sites and other mental health service user networks. They will work together with the wider University research team (which also includes a lived experience researcher leading on the qualitative work), to recruit study participants, conduct semi-structured telephone/online interviews with up to 50 service users, and carers, and contribute to the coding and analysis of the qualitative data. The lived experience researchers will be provided with qualitative methods training beforehand, covering good practice in conducting interviews. The qualitative interviews will explore the impact of COVID-19 and ethnic inequalities, for people living with multiple long-term health conditions and mental health problems, in accessing or experiencing barriers to care, discrimination within health services and health-seeking, which will shed light on potential mechanisms.

Participants will be purposively sampled from three sites across England (Lambeth (London), Manchester and Birmingham & Solihull), by ethnicity, mental health diagnoses and physical health comorbidities. These sites have been specifically selected as they represent areas of high ethnic diversity which have also had high rates of COVID-19 infection, and because they are pilot sites for the Patient and Carer Race Equality Framework (PCREF, described below). The main ethnic groups to be purposively sampled across sites will be people of Black Caribbean, Black African, Indian, Pakistani and Bangladeshi descent. Recruitment to the qualitative study will be supported through local community organisation networks/ charities as well as social media outlets. Participants will be offered the option of doing interviews either through video or audio online conferencing on Microsoft Teams or Zoom or will be able to phone into these platforms on a 0800 freephone number.

Topic guides for the interviews will be developed following consultation with the study steering group and designed to assess the experiences of people living with severe mental illnesses and other long-term conditions, and their carers, of the COVID-19 pandemic. In particular, areas for enquiry will include the impact of COVID-19 on living with long-term mental and physical health conditions, the impact of COVID-19 on being able to access services (including access to remote healthcare services), public health messages and information/advice received relating to COVID-19, barriers to accessing services, social support and networks during the pandemic, experiences of racism and discrimination when trying to access services during the pandemic, impact of possible guidance to shield, impact of COVID-19 interventions (mask wearing, social distancing and hand washing), perceptions of vaccination and opportunities for involvement in improvement initiatives.

Interviews will be de-identified, transcribed and imported into N-Vivo. Thematic analyses will be used. A triangulation protocol will be devised to identify meta-themes and synthesise and used to integrate across qualitative/quantitative findings, illuminating areas of convergence or discrepancy. Qualitative insights may inform quantitative study design/ analysis, for example by informing variable selection for models and potentially inform the interpretation of quantitative findings. Qualitative findings may also lead to the generation of further hypotheses which could be used to inform future quantitative analyses. Qualitative findings may also highlight barriers to accessing services from the perspectives of service users (e.g. access, discrimination, stigma, fear of using services, involvement in improvement initiatives). These will inform interpretation and development of actionable recommendations. These recommendations will also be shaped by the links with the development of the PCREF in the case study sites.

### Quantitative results

**Fig. 1:**
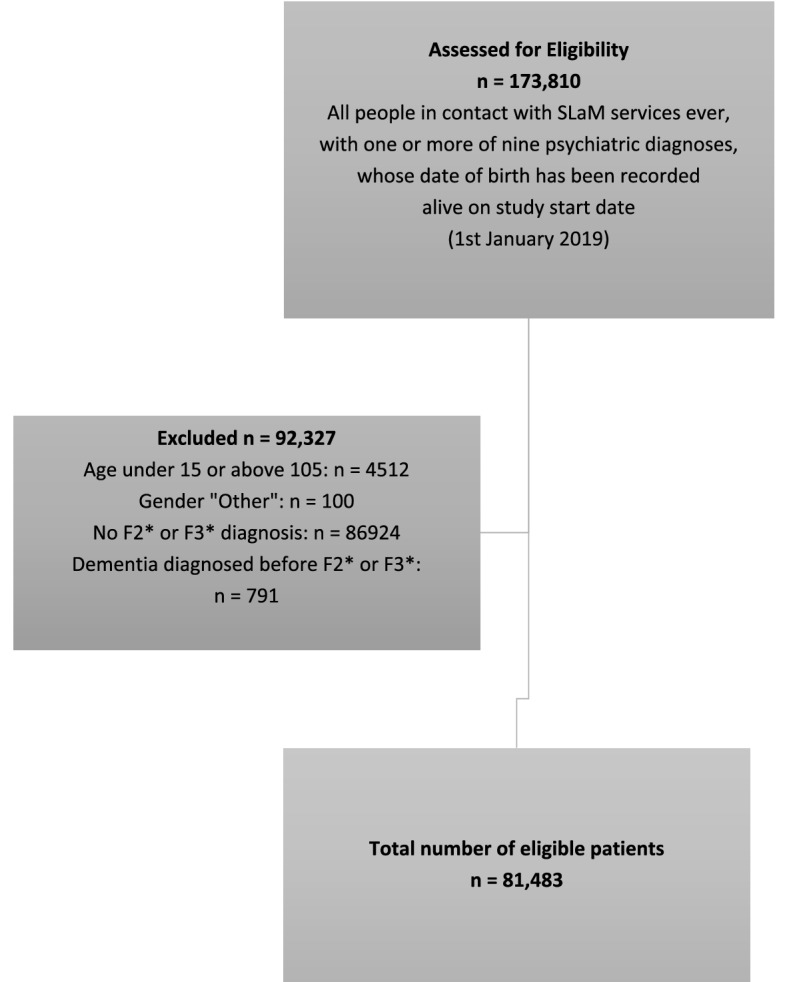
Flowchart for study sample—CRIS

**Fig. 2:**
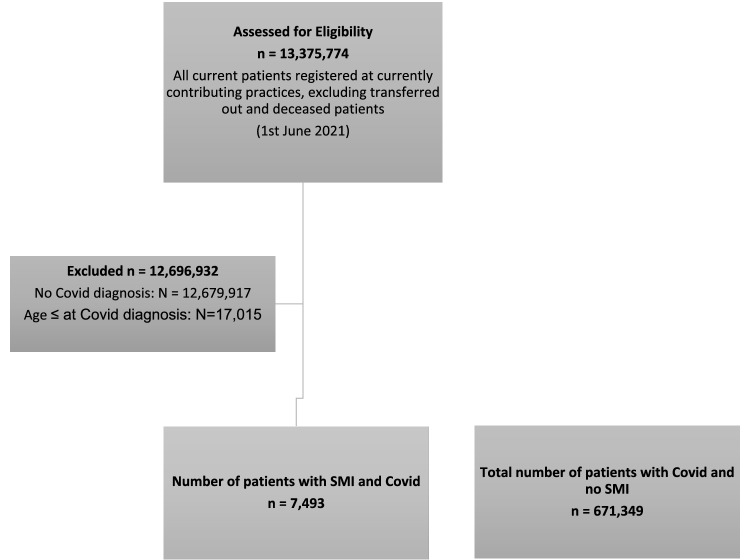
Flowchart for study sample—CPRD

Figures 1 and 2 provide an overview of samples for CRIS and CPRD. Table 1 provides a descriptive overview of CRIS, highlighting social and demographic indicators of people as identified as having either an ICD-10 F2* (schizophrenia-spectrum) or F3* (affective disorders, including bipolar affective disorders diagnoses), by ethnicity. Deaths over the observation period (from 1st January 2019 until 7th October 2021, including from COVID-19 are also displayed. In general, people within the sample experienced high levels of relationship disruption or were likely to be single, most of the sample resided in areas of higher deprivation and were living with more than one psychiatric comorbidity. Overall, 4% of the sample (*N *= 3292) died OVER the observation window.

**Table 1:** Demographic overview of the CRIS sample

	White British (*N *= 32,324)	Irish (*N *= 1401)	Black Caribbean (*N *= 10,466)	Black African (*N *= 6473)	Pakistani (*N *= 667)	Bangladeshi (*N *= 403)	Indian (*N *= 1256)	Other (*N *= 826)	Missing (*N *= 12,406)	Overall (*N *= 81,483)	
Age											
Mean (SD)	48.0 (17.9)	56.0 (17.3)	44.4 (17.1)	44.6 (14.5)	44.5 (16.4)	42.1 (15.4)	47.8 (18.3)	37.7 (15.0)	43.7 (16.1)	46.1 (16.9)	*p *< 0.001
Gender											
Female	17,716 (54.8%)	695 (49.6%)	5708 (54.5%)	3,557 (55.0%)	375 (56.2%)	242 (60.0%)	713 (56.8%)	468 (56.7%)	6,727 (54.2%)	44,594 (54.7%)	*p *= 0.002
Male	14,608 (45.2%)	706 (50.4%)	4758 (45.5%)	2,916 (45.0%)	292 (43.8%)	161 (40.0%)	543 (43.2%)	358 (43.3%)	5,679 (45.8%)	36,889 (45.3%)	
Relationship status											
Married/civil partner/cohabiting	5324 (16.5%)	216 (15.4%)	834 (8.0%)	998 (15.4%)	224 (33.6%)	121 (30.0%)	321 (25.6%)	96 (11.6%)	483 (3.9%)	11,489 (14.1%)	*p *< 0.001
Single	17,673 (54.7%)	732 (52.2%)	7110 (67.9%)	3,467 (53.6%)	239 (35.8%)	156 (38.7%)	563 (44.8%)	474 (57.4%)	1,416 (11.4%)	39,669 (48.7%)	
Divorced/separated/widowed	3455 (10.7%)	261 (18.6%)	880 (8.4%)	874 (13.5%)	83 (12.4%)	53 (13.2%)	136 (10.8%)	44 (5.3%)	188 (1.5%)	7,410 (9.1%)	
Area level deprivation											
Median [Q1, Q3]	10,500 [5880, 16,600]	8,140 [5450, 13,500]	7,440 [5050, 12,100]	6,870 [4790, 11,000]	10,500 [6340, 14,600]	6,530 [4570, 10,400]	10,700 [6420, 15,400]	8,590 [5320, 13,700]	9,710 [5720, 15,400]	9,030 [5460, 14,400]	*p *< 0.001
*N*. of psychiatric conditions											
Mean (SD)	11.0 (1.02)	11.0 (1.08)	11.0 (1.03)	10.7 (0.887)	10.7 (0.930)	10.6 (0.812)	10.9 (0.978)	11.0 (1.06)	10.4 (0.677)	10.8 (0.959)	*p *< 0.001
Deaths											
Alive	30,490 (94.3%)	1,285 (91.7%)	10,092 (96.4%)	6,328 (97.8%)	646 (96.9%)	394 (97.8%)	1,189 (94.7%)	810 (98.1%)	12,048 (97.1%)	78,191 (96.0%)	*p *< 0.001
Dead	1,834 (5.7%)	116 (8.3%)	374 (3.6%)	145 (2.2%)	21 (3.1%)	< 10 (2.2%)	67 (5.3%)	16 (1.9%)	358 (2.9%)	3,292 (4.0%)	
Schizophrenia-spectrum disorders											
Not diagnosed	24,139 (74.7%)	986 (70.4%)	4,987 (47.6%)	3,064 (47.3%)	420 (63.0%)	261 (64.8%)	792 (63.1%)	549 (66.5%)	9,465 (76.3%)	55,250 (67.8%)	*p *< 0.001
Diagnosed	8,185 (25.3%)	415 (29.6%)	5,479 (52.4%)	3,409 (52.7%)	247 (37.0%)	142 (35.2%)	464 (36.9%)	277 (33.5%)	2,941 (23.7%)	26,233 (32.2%)	
Affective disorders											
Not diagnosed	4,724 (14.6%)	236 (16.8%)	3,297 (31.5%)	2,101 (32.5%)	143 (21.4%)	93 (23.1%)	282 (22.5%)	151 (18.3%)	2,367 (19.1%)	16,287 (20.0%)	*p *< 0.001
Diagnosed	27,600 (85.4%)	1,165 (83.2%)	7,169 (68.5%)	4,372 (67.5%)	524 (78.6%)	310 (76.9%)	974 (77.5%)	675 (81.7%)	10,039 (80.9%)	65,196 (80.0%)	

Key: Deaths from 1^st^ January 2019 until 5th May 2021. Area Level Deprivation: The Index of Multiple Deprivation (IMD) is divided into 10 ordered groups, where: Ranks 1 to 3,284 represent the 10% most deprived areas, while Ranks 29,560 to 32,844 represent the 10% least deprived areas. The sample is associated to a median value below 10,700 (30% to 40% most deprived areas). Chi squared tests used to derive *p* values for categorical variables and one way analysis of variance (ANOVA) for continuous variables

Table 2 displays the presence of psychiatric comorbidities in the CRIS sample, by ethnicity. Across the sample, the most common psychiatric comorbidities in people with schizophrenia-spectrum and affective disorders were somatoform/anxiety disorders (30.2% of the sample) and substance use disorders (14.1%), as well as personality disorders (12.2%).

**Table 2:** Comorbid psychiatric conditions (‘psychiatric multimorbidities’) in people with schizophrenia-spectrum and affective disorders (CRIS sample)

	White British (*N *= 32,324)	Irish (*N *= 1401)	Black Caribbean (*N *= 10,466)	Black African (*N *= 6473)	Pakistani (*N *= 667)	Bangladeshi (*N *= 403)	Indian (*N *= 1256)	Other (*N *= 826)	Missing (*N *= 12,406)	Overall (*N *= 81,483)
Anxiety/somatoform disorders										
Not diagnosed	20,944 (64.8%)	930 (66.4%)	7689 (73.5%)	4683 (72.3%)	472 (70.8%)	297 (73.7%)	871 (69.3%)	514 (62.2%)	9749 (78.6%)	56,877 (69.8%)
Diagnosed	11,380 (35.2%)	471 (33.6%)	2777 (26.5%)	1790 (27.7%)	195 (29.2%)	106 (26.3%)	385 (30.7%)	312 (37.8%)	2657 (21.4%)	24,606 (30.2%)
Eating disorders										
Not diagnosed	30,672 (94.9%)	1367 (97.6%)	10,198 (97.4%)	6375 (98.5%)	640 (96.0%)	393 (97.5%)	1184 (94.3%)	776 (93.9%)	12,208 (98.4%)	78,628 (96.5%)
Diagnosed	1652 (5.1%)	34 (2.4%)	268 (2.6%)	98 (1.5%)	27 (4.0%)	10 (2.5%)	72 (5.7%)	50 (6.1%)	198 (1.6%)	2855 (3.5%)
Pervasive developmental disorders										
Not diagnosed	30,473 (94.3%)	1362 (97.2%)	9793 (93.6%)	6286 (97.1%)	637 (95.5%)	385 (95.5%)	1179 (93.9%)	758 (91.8%)	12,064 (97.2%)	77,797 (95.5%)
Diagnosed	1851 (5.7%)	39 (2.8%)	673 (6.4%)	187 (2.9%)	30 (4.5%)	18 (4.5%)	77 (6.1%)	68 (8.2%)	342 (2.8%)	3686 (4.5%)
Learning disorders										
Not diagnosed	31,354 (97.0%)	1367 (97.6%)	9954 (95.1%)	6303 (97.4%)	655 (98.2%)	390 (96.8%)	1207 (96.1%)	791 (95.8%)	12,299 (99.1%)	79,339 (97.4%)
Diagnosed	970 (3.0%)	34 (2.4%)	512 (4.9%)	170 (2.6%)	12 (1.8%)	13 (3.2%)	49 (3.9%)	35 (4.2%)	107 (0.9%)	2144 (2.6%)
Personality disorders										
Not diagnosed	26,994 (83.5%)	1173 (83.7%)	9088 (86.8%)	6048 (93.4%)	612 (91.8%)	376 (93.3%)	1112 (88.5%)	711 (86.1%)	11,621 (93.7%)	71,570 (87.8%)
Diagnosed	5330 (16.5%)	228 (16.3%)	1378 (13.2%)	425 (6.6%)	55 (8.2%)	27 (6.7%)	144 (11.5%)	115 (13.9%)	785 (6.3%)	9913 (12.2%)
Dementia										
Not diagnosed	31,557 (97.6%)	1333 (95.1%)	10,186 (97.3%)	6394 (98.8%)	655 (98.2%)	397 (98.5%)	1216 (96.8%)	821 (99.4%)	12,354 (99.6%)	79,953 (98.1%)
Diagnosed	767 (2.4%)	68 (4.9%)	280 (2.7%)	79 (1.2%)	12 (1.8%)	< 10 (1.5%)	40 (3.2%)	< 10 (0.6%)	52 (0.4%)	1530 (1.9%)
Substance use disorders										
Not diagnosed	26,751 (82.8%)	1012 (72.2%)	8377 (80.0%)	5698 (88.0%)	620 (93.0%)	375 (93.1%)	1113 (88.6%)	715 (86.6%)	11,731 (94.6%)	70,031 (85.9%)
Diagnosed	5573 (17.2%)	389 (27.8%)	2089 (20.0%)	775 (12.0%)	47 (7.0%)	28 (6.9%)	143 (11.4%)	111 (13.4%)	675 (5.4%)	11,452 (14.1%)

*p *< 0.001 for all displayed variables, derived from Chi squared tests

Table 3 displays the demographic overview of the CPRD sample. In the CPRD sample, people living with severe mental illnesses were older, more likely to be male, with similar geographical distribution of residence t people without a severe mental illness. Deaths were notably higher in people with severe mental illnesses (10%) compared to the control sample (2%) and multimorbidity conditions more prevalent.

**Table 3:** Demographic characteristics of the CPRD sample

	Severe mental illnesses^a^ (*N *= 7493, 1.1%)	No severe mental illness (*N *= 671,349, 98.9%)
Age (years) mean (SD)	55.4 (19.6)	41.4 (20.9)
Gender		
Female	4200 (56)	369,721 (55)
Male	3293 (44)	301,621 (45)
Ethnicity		
White British	2194 (29)	164,047 (24)
Black	387 (5)	23,252 (3)
South Asian	540 (7)	59,241 (9)
Mixed	99 (1)	8,030 (1)
Other	2850 (38)	240,331 (36)
Unknown/missing	1423 (19)	176,448 (26)
Deprivation—quintiles		
First	842 (11)	111,657 (17)
Second	1,125 (15)	116,027 (17)
Third	1,259 (17)	119,949 (18)
Fourth	1647 (22)	143,660 (21)
Fifth	2177 (29)	151,558 (23)
Missing	443 (6)	28,498 (4)
English regions		
North East	284 (4)	22,734 (3)
North West	1658 (22)	143,971 (21)
Yorkshire	189 (3)	19,353 (3)
East Midlands	139 (2)	17,391 (3)
West Midlands	1153 (15)	109,581 (16)
East Anglia	233 (3)	23,779 (4)
South West	619 (8)	55,404 (8)
South Central	763 (10)	64,147 (10)
London	1826 (24)	157,869 (24)
South East	607 (8)	55,864 (8)
Northern Ireland	15 (0)	832 (0)
Missing	7 (0)	424 (0)
Deaths	660 (9)	16,519 (2)
Annual consultations	1.49 (1.2)	1.39 (0.5)
Multimorbidity median (IQR)	4 (3.5)	3 (2.4)
0/1 conditions	1456 (20)	416,865 (62)
2 conditions	1449 (19)	105,882 (16)
3 conditions	1410 (19)	63,163 (9)
4 conditions	1118 (15)	35,683 (5)
5 conditions	772 (10)	20,863 (3)
6 + conditions	1288 (17)	28,893 (4)

^a^Defined as schizophrenia-spectrum and bipolar affective disorders and non-organic psychoses. *p* value for all variables *p *< 0.001 except gender which was *p *= 0.151, Chi squared tests

## Discussion

The advent of COVID-19 in early 2020 brought to the surface and magnified pre-existing health inequalities, particularly known to impact minority ethnic groups in the UK and in people living with pre-existing severe mental health problems with other longstanding health conditions [2–4]. Research with a specific focus on the impact of the pandemic on people living with multimorbidities and severe mental disorders has remained scarce, particularly with a focus on intersections with ethnicity. Quantitative analyses will provide an overview of trends, both nationally and in urban catchment areas, and will shed light on patterns of inequalities and modifiable risks which may underlie these. However, to get ‘under the skin’ of the data, this study will also employ qualitative methods, co-produced with peer researchers, to further shed light on the experiences of those most impacted by the pandemic. Our close relationship with the PCREF, a national framework to address race equality in mental health, may ensure an actionable focus to study findings.

The strengths of the project reside in its combination of methodologies, which synthesise quantitative results with qualitative perspectives. Our quantitative strategy will provide both a national perspective with CPRD primary care data (aiming for generalizability) as well as a more focused investigation with secondary mental healthcare services data from an ethnically diverse catchment area where there have been high levels of COVID-19 infections and deaths [28]. A further strength of the study will be in the use of qualitative methodologies which will include recruiting and working with peer researchers with lived experience, who will contribute to qualitative data collection and analysis. The use of a participatory action research approach will enhance the ability to co-produce research findings, with a view to developing actionable recommendations. A limitation of our approaches is that the data sources underpinning quantitative analyses are routinely generated health records data, and detail on important experiences such as individual-level socioeconomic position, education as well as COVID-19 related job loss and bereavements are currently unavailable within these health records. Although these important mediators for potential outcomes cannot be assessed in the quantitative analyses, we anticipate that the qualitative interviews may shed light on some of these experiences. A further limitation is that we may need to aggregate some of the smaller minority ethnic groups in the sample, due to lower statistical power which may hamper analyses. This will mask heterogeneity and differences in experiences between groups. Finally, for analyses using CPRD, cause-specific mortality data is not available in this study. Therefore, the relationships between COVID-19 infection and other causes of death (for example by suicide or other somatic illnesses) will not be discernible through these analyses.

The process of synthesising across different types of data sources (e.g. quantitative data and qualitative interviews) as well as across two different datasets (one reflecting primary care the other secondary mental healthcare) will present challenges. Within the research team, we will discuss and develop methods to deal with this issue. A final challenge has been the pandemic situation; COVID-19 continues to be in a state of flux; our strategy for data collection and analyses has needed to be flexible to potentially incorporate new data and themes as the pandemic, and its sequelae, continues to unfold.

We envisage that the study findings will result in actionable recommendations to enhance patient safety and may lead to recommendations which minimise ethnic inequalities. Findings will be relayed to public health commissioners, and the NHS-England mental health equalities taskforce, as well as to other stakeholders involved in mental health equalities in England using a range of media including lay summaries, blog posts and reports. If appropriate, statistical code will be deposited in open-source repositories such as github. Visualisation techniques may be used to make findings more accessible and may assist with further dissemination of findings.

In conclusion, the COVID-19 pandemic has led to wide ranging impacts on people living with severe mental health conditions, potentially further impacted by the presence of other psychiatric and physical health multimorbidites, with a concern around ethnic inequalities being further exacerbated. In this study, we anticipate rapid findings which will lead to actionable recommendations, enhanced by the mixed methods approaches employing large-scale data analytics (combining epidemiological and data science methodologies) with insights from qualitative and analysis, co-produced with people with lived experience.

## Data Availability

Data are owned by a 3rd party SLaM BRC CRIS tool which provides access to anonymised data derived from SLaM electronic medical records. These data can only be accessed by permitted individuals from within a secure firewall (i.e. remote access is not possible and the data cannot be sent elsewhere) in the same manner as the authors.

## Footnotes

1 Myocardial infarction, heart disease, stroke/cerebral infarction, venous thromboembolism, arrhythmia, atrial fibrillation, atherosclerosis, hypertension, type I and type II diabetes, CKD, COPD, asthma, non-alcoholic liver disease, alcoholic liver disease, rheumatoid arthritis, lupus, psoriasis, IBS, Crohn’s disease, ulcerative colitis, systemic vasculitis, pancreatitis, stomach ulcers, gastrointestinal, periodontal disease, pulmonary hypertensive disease, dementia (vascular, Alzheimer’s, Lewy body frontotemporal), depression, anxiety, bipolar, schizophrenia, eating disorders, ADHD, autism, epilepsy, PTSD, substance abuse, HIV, osteoporosis, autoimmune disorders.
